# Continuous Renal Replacement Therapy after Liver Transplantation: Peri-Operative Associated Factors and Impact on Survival

**DOI:** 10.3390/jcm11133803

**Published:** 2022-06-30

**Authors:** Gennaro Martucci, Matteo Rossetti, Sergio Li Petri, Rossella Alduino, Riccardo Volpes, Giovanna Panarello, Salvatore Gruttadauria, Gaetano Burgio, Antonio Arcadipane

**Affiliations:** 1 Department of Anesthesia and Intensive Care, IRCCS-ISMETT (Istituto Mediterraneo per I Trapianti e Terapia ad Alta Specializzazione), Via Tricomi 5, 90133 Palermo, Italy; gmartucci@ismett.edu (G.M.); mrossetti@ismett.edu (M.R.); gpanarello@ismett.edu (G.P.); aarcadipane@ismett.edu (A.A.); 2 Abdominal Surgery and Organ Transplantation Unit, Department for the Treatment and Study of Abdominal Diseases and Abdominal Transplantation, IRCCS-ISMETT (Istituto Mediterraneo per I Trapianti e Terapia ad Alta Specializzazione), 90133 Palermo, Italy; slipetri@ismett.edu (S.L.P.); sgruttadauria@ismett.edu (S.G.); 3 Research Office, IRCCS-ISMETT (Istituto Mediterraneo per I Trapianti e Terapia ad Alta Specializzazione), 90133 Palermo, Italy; ralduino@ismett.edu; 4 Hepatology Unit, Department for the Treatment and Study of Abdominal Diseases and Abdominal Transplantation, IRCCS-ISMETT (Istituto Mediterraneo per I Trapianti e Terapia ad Alta Specializzazione), 90133 Palermo, Italy; rvolpes@ismett.edu

**Keywords:** liver transplantation, veno-venous bypass, transfusion, creatinine, acute kidney injury

## Abstract

Continuous renal replacement therapy (CRRT) following orthotopic liver transplantation (OLT) is usually started for multifactorial reasons, with variable incidence among series. This paper presents a single-center retrospective observational study on the early use (within one week) of CRRT after consecutive cadaveric OLT from January 2008 to December 2016. Preoperative patient characteristics and intraoperative data were collected, and patients were divided into two groups (CRRT and no CRRT) to explore the factors associated with the use of CRRT. Repeated measurements of postoperative creatinine were analyzed with generalized estimating equation (GEE) models. Among 528 OLT patients, 75 (14.2%) were treated with CRRT at least once in the first week. Patients treated with CRRT showed lower survival in a Kaplan–Meier curve (log-rank *p* value < 0.01). Patients treated with CRRT had a more severe preoperative profile, with a significantly higher age, MELD, BUN, creatinine, and total bilirubin, as well as a longer surgery time and a higher number of transfusions of red blood cells, plasma, and platelets (all *p* values < 0.05). In a stepwise multiple analysis, the following characteristics remained independently associated with the use of CRRT: the MELD score OR 1.12 (95% CL: 1.07–1.16), *p* value < 0.001, and the preoperative value for blood urea nitrogen OR 1.016 (95% CL: 1.010–1.023), *p* value < 0.001. The early use of CRRT after OLT occurred at a low rate in this large cohort; however, it was associated with worse outcomes. Apart from the preoperative severity, repeated intraoperative hypotension episodes, which were likely modifiable or preventable, were associated with the increased use of CRRT and higher postoperative creatinine.

## 1. Introduction

Acute kidney injury (AKI) is common following orthotopic liver transplantation (OLT), with an incidence ranging from 17% to 64% [1] according to the literature, and an 8–17% [2] need for renal replacement therapy, contributing to a prolonged hospital stay [3], infectious complications, and a reduction in patient survival [4,5,6].

In addition to these possible complications, sepsis occurs more frequently in patients treated with continuous renal replacement therapy (CRRT) [7]. This can lead to the augmentation of kidney injury via cytokine storm, causing hypotension and renal ischemia [8].

The etiology of AKI is widely multifactorial, and includes pre-existing factors (e.g., renal dysfunction of hepatorenal syndrome, hyperbilirubinemia or hypoproteinemia, and hyponatremia), intraoperative factors (such as hypotension, the volume of transfused blood products, and surgical technique) [9], and postoperative factors (acute tubular necrosis, post-reperfusion syndrome, graft dysfunction, drug-induced interstitial nephritis, and conditions of polypharmacological etiology) [10].

The use of calcineurin inhibitors during the early postoperative hospital stay can result in the derangement of renal function, and contribute to this picture [11,12]. Moreover, the use of CRRT in the early postoperative period is also prompted by the need to avoid fluid overload, which seems to be a relevant cause of the increase in ischemia-reperfusion injury and can worsen the graft perfusion [13].

The use of CRRT after OLT has been associated with worse outcomes and increased mortality. During the periods when the patient is on the waiting list and in intra-operatory management, the factors indicating the need for CRRT, which are potentially also modifiable, are still unclear and need to be elucidated in order to arrange the best anesthesiology care [4,12].

Among the possible factors contributing to AKI after OLT, intraoperative hypotension has been deemed to be a relevant cause. However, the optimal ways of managing hypotension during surgery—vasopressor administration, fluid balance, and blood product transfusion—are still left to the discretion of the single center or anesthesiologist, without clear guidelines [14]. For the purpose of avoiding splanchnic congestion, many authors have suggested the intraoperative use of a veno-venous bypass, although its use, despite being pathophysiologically sound, is still criticized and does not rely on solid evidence [15,16].

The purpose of the present study was to describe the use of CRRT in a large cohort of OLT recipients in the early period after transplantation, and to explore the preoperative and intraoperative factors associated with its use, as well as the impact of such a support therapy on mortality after OLT.

## 2. Materials and Methods

In this single-center study, we retrospectively collected data from consecutive patients undergoing cadaveric OLT at IRCCS-ISMETT (Istituto Mediterraneo per i Trapianti e Terapie ad Alta Specializzazione, Palermo, Italy) from January 2008 to December 2016. The study was approved by the ISMETT Ethics Committee IRRB/6/13.

### 2.1. Data Collection

Data including demographics, laboratory tests, and intraoperative and postoperative parameters were obtained through an automated query from electronic medical records (Sunrise Clinical Manager, Allscripts Healthcare Solutions, Inc., Chicago, IL, USA).

Preoperative variables included age, gender, and type of liver disease (divided into 3 classes identified as cirrhosis, acute liver failure, and hepatocellular carcinoma (HCC)). Predisposing factors included body mass index (BMI), Model for End-stage Liver Disease (MELD) score, pre-existing comorbidities, renal function (blood urea nitrogen (BUN), and creatinine.

Intraoperative parameters included hemodynamics, surgical times, and rate of transfusions. For hemodynamics, heart rate, invasive arterial pressure, peripheral arterial hemoglobin saturation, and the use and dose of vasoactive agents were recorded every 5 min, and diuretic output was updated every hour to easily obtain fluid balance at the end of the intervention. Hypotensive episodes occurring during the intraoperative period were defined as 3 consecutive registered mean invasive arterial pressure detections that were lower than 65 mmHg (which equates to a hypotensive event presenting for at least 15 min). Therefore, each patient could have presented with either no or one or more hypotensive events, depending on intraoperative dynamics. The use of vasoactive drugs and the dose were also collected. At our institution, hemodynamic monitoring during OLT is performed in all recipients by two invasive arterial lines (radial and femoral artery), central venous pressure, and trans-esophageal echocardiography. A pulmonary artery catheter was used for each patient with risk factors for pulmonary hypertension, and in every case of increased estimated pulmonary pressure at the trans-thoracic echocardiography performed during the work-up for listing.

The use of femoral-to-jugular veno-venous bypass (VVB) and the duration of surgery were also reported. Transfusions were reported as the number of patients transfused and the amount of transfusion of packed red blood cells (PRBC), plasma, and platelets.

Postoperative data were recorded daily for 7 days after OLT; these were creatinine and the Modification of Diet in Renal Disease (MDRD) study equation for estimating glomerular filtration rate. CRRT use was also reported if CRRT was employed at least once during the 7 days following the intervention.

The follow-up lasted one year after transplantation, and the date of death was reported to evaluate survival after OLT.

### 2.2. Statistical Analysis

Continuous and categorical variables are expressed as median with interquartile range and as frequency with percentage, respectively. To compare continuous and categorical variables among groups, Wilcoxon tests and chi-square tests were used, respectively. To explore the differences between the recipients (preoperative risk factors) in predicting events, logistic regression and the Kaplan–Meier survival method were used when appropriate. The log-rank test was used to compare Kaplan–Meier survival curves.

Given the presence of repeated measurements, generalized estimating equation (GEE) models were applied to evaluate the effect of time on the outcome. For the estimation of the model parameters, correlations between the repeated measures of the outcome variable were considered. The analyses were performed using an exchangeable correlation working matrix. The matrices approaches were chosen based on the quasi-likelihood under the independence model criterion (QIC) obtained. Levels of significance were set at a *p* < 0.05. Statistical analyses were performed using SAS software Version 9.4 (SAS Institute Inc., Cary, NC, USA).

## 3. Results

### 3.1. Patient Characteristics and Differences among Patients on CRRT or Not on CRRT in the First Postoperative Week

From January 2008 to December 2016, 528 cadaveric OLTs were performed at our institute. The preoperative characteristics of patients are summarized in Table 1. Among the entire cohort, 75 patients (14.2%) were supported with CRRT at least once in the first week after OLT. In this subgroup undergoing CRRT, patients were on renal support for a median of 4 days (95% confidence interval 2–6). First, we compared the preoperative and intraoperative characteristics of the two study groups: patients treated with CRRT or without CRRT in the first week after OLT (Table 1). Patients treated with CRRT had a more severe preoperative profile, with a significantly higher age, and higher MELD, BUN, creatinine, and total bilirubin (all *p* values < 0.05). Patients on CRRT after OLT also had a more complex intraoperative period because they had a longer surgery. Patients on CRRT also had a higher number of transfusions, and a higher quantity of blood products transfused (higher transfusion of packed red blood cells, plasma, and platelets). As expected, given these preoperative and intraoperative differences, the outcomes were also worse in the CRRT group. The intensive care unit (ICU) length of stay (LOS), as well as the hospital LOS, were higher, as was the percentage of patients who died in the first year after OLT.

### 3.2. Preoperative and Intraoperative Factors Associated with the Use of Bypass Technique

Since VVB is considered to be a technique for reducing kidney damage during transplantation, before testing the association of bypass with the use of CRRT, we explored the factors prompting the use of bypass intraoperatively. Considering the preoperative patient data, the factors favoring the use of intraoperative bypass were higher age, MELD, BUN, MDRD, and total bilirubin. Moreover, the intraoperative hemodynamic instability (odds ratio (OR) 95% confidence interval (CI) 1.022 [1.005–1.04] *p* value = 0.011) and the transfusion of plasma were both associated with the use of bypass. A complete logistic regression analysis with all the preoperative factors and intraoperative factors is presented in Table 2.

In a stepwise multiple analysis of those statistically significant variables on the univariate analysis, three factors remained independently significant: age OR (95% CI) 1.019 (1.002–1.036) *p* value = 0.0283, BUN OR (95% CI) 1.007 (1.001–1.013) *p* value = 0.0209, and intraoperative hypotension episodes OR (95% CI) 1.028 (1.008–1.048) *p* value = 0.0256.

### 3.3. Peri-Operative Factors Associated with the Use of CRRT

To explore the factors associated with the use of CRRT after OLT, we carried out several univariate logistic regressions of a variety of predictive factors and the use of CRRT. In these analyses, the presence of hepatocarcinoma was associated with less postoperative use of CRRT, while, as expected, acute liver failure was strongly associated with the use of CRRT. As intraoperative factors, hypotension and transfusions were also associated with the use of CRRT. A complete table of OR for predictive variables is presented in Table 3.

In a stepwise multiple analysis of those variables that were statistically significant on univariate analysis, only two preoperative factors remained independently associated with the use of postoperative CRRT: the MELD score OR 1.12 (95% CL: 1.07–1.16), *p* value < 0.001, and the preoperative value for BUN OR 1.016 (95% CL: 1.010–1.023), *p* value < 0.001.

### 3.4. The Impact of CRRT on Mortality after OLT

**Figure 1:**
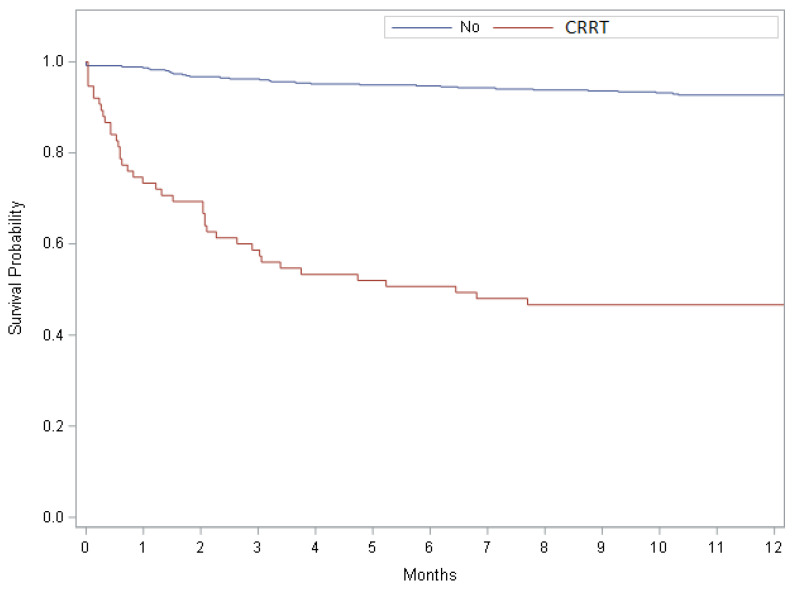
Kaplan–Meier curve showing survival between the two groups (continuous renal replacement therapy (CRRT) in the first week after transplant vs. no CRRT).

Overall, at the one-year follow-up, 73 of the 528 patients had died. As shown by the Kaplan–Meier curve (log-rank *p* value < 0.01), survival in the week immediately following transplantation favored the group without CRRT (Figure 1).

### 3.5. Postoperative Longitudinal Evolution of Creatinine

**Figure 2:**
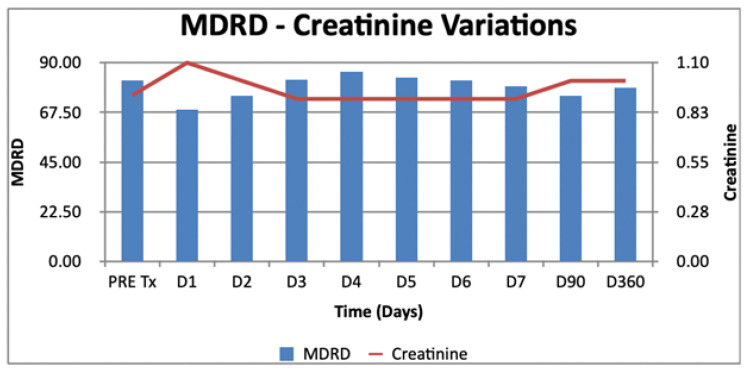
Evolution of creatinine and MDRD in the postoperative period.

Moreover, we explored the evolution of creatinine and MDRD in the early postoperative period, as shown in Figure 2.

We explored the factors associated with changes in creatinine over the follow-up period using several generalized estimate equation models, adjusting each analysis for the preoperative creatinine (Table 4).

In a longer follow-up, at 30 days after OLT, 29 (5.7%) out of 513 patients were still on intermittent hemodialysis; at one year after OLT, 7 (1.6%) out of 439 patients were on hemodialysis.

## 4. Discussion

This retrospective analysis of a single-center cohort of 528 OLT patients has shown that CRRT was started in the first week after transplantation in 14% of cases, and, in those patients, CRRT use was associated with a higher mortality at one year. Preoperative risk factors for the use of CRRT were preoperative renal function, age, and the severity of the disease (acute liver failure, MELD, and the presence of hepatocarcinoma). Intraoperative factors associated with the use of CRRT were likely related to the complexity of the procedure (the length of surgery, the number of transfusions, and hemodynamic instability).

CRRT after OLT has been applied at varying rates, ranging from 7% to 40% of cases [12]. Regarding this question, the timing is still undefined due to a lack of compelling evidence. A recent cohort study examining general considerations for critically ill patients recognized a faster recovery for patients with the early use of CRRT; however, in this case, the main indication was acute kidney injury, but the use of CRRT may also be indicated by fluid overload, infection, and accumulation of toxins. In our cohort, 14.2% of patients were treated early with CRRT, and the main result of the stepwise multiple analysis was that the severity of the patient (through the MELD score) and the preoperative BUN were identified as risk factors. Moreover, the use of CRRT in these patients was substantial, indicating that, when applied, CRRT was adopted for several days during the first week. These reasonable factors were confirmed by a recent study on the recovery of kidney function after OLT [12].

The main observation in our cohort was that CRRT was strongly associated with mortality, and this concept should foster the notion that several strategies should be implemented to reduce metabolic derangement in the peri-operative period, and adequate prediction should be put in place [14,17]. This is not a new concept, since the association between acute kidney failure and OLT has previously been highlighted in the literature. However, the importance of intraoperative factors, which are potentially modifiable, should also be strongly emphasized to evaluate the prognosis and to improve the strategies of care.** Despite evidence, hypotension in modern anesthesia for OLT, characterized by shorter surgical times and a reduced number of transfusions, is still a principal determinant of postoperative complications [18].

The use of VVB was introduced to increase the tolerance of caval cross-clamping in older or particularly severe patients. As a main protective factor, VVB is believed to reduce splanchnic congestion and, consequently, the renal flow by reducing the cardiac filling, thus stabilizing the hemodynamic picture and preventing metabolic decompensation [19]. Still, the most recent literature suggests using bypass in select fragile patients to reduce renal complications or to facilitate complex surgery, with much evidence of feasible OLT without VVB [20]. In our cohort, VVB was applied to 16.9% of patients; as expected in a limited number of patients, the factors more closely associated with the use of bypass confirm the current orientation. The more fragile patients were represented by age. Consequently, the older the patient, the more the patient was supported intraoperatively by VVB, with the aim of protecting the patient from metabolic derangement. Despite what has been claimed, apart from the effect on the surgical activity and duration, the utility of VVB should likely be further verified regarding ischemia-reperfusion injury, a still underexplored topic, with recent advancements on marginal graft donors performed either with or without VVB.

This study has several limitations. Firstly, it was a retrospective, single-center study, and there is need for further prospective studies to address the question of CRRT after OLT. Moreover, the use of CRRT after OLT may have overlapped with acute postoperative kidney failure and fluid overload. Despite this limitation, the idea that different indications after OLT likely often coexist should not be neglected. In light of this, the absence of clear reasons for starting CRRT is a concrete limitation. On the other hand, this is a large cohort, and the described scenario is a concrete real-time picture, where, as witnessed by the low average level of creatinine, the renal failure is multifactorial and not merely based on standard criteria. The retrospective data collection was performed over a long period of time; therefore, even though the approach of the team did not change during these years, it might be biased by the changing of practices and personnel. However, the data collection was robust and automated through a complete online medical chart.

## 5. Conclusions

CRRT after OLT is still frequently needed and is associated with high mortality rates in patients with severe preoperative conditions. Among the modifiable risk factors, hemodynamic instability seems to play a relevant role, though further research is needed to understand potential protective strategies to preserve renal function.

## Figures and Tables

**Table 1:** Comparison of the preoperative and intraoperative characteristics of the two study groups: patients treated with CRRT or without CRRT in the first week after OLT. Preoperative and intraoperative variables, as well as outcomes, are shown. BMI: Body mass index; CRRT: continuous renal replacement therapy; MELD: Model for End-stage Liver Disease; MDRD: Modification of Diet in Renal Disease; PRBC: packed red blood cells; ICU: intensive care unit.

	Overall *n* = 528	CRRT *n* = 75	NO CRRT *n* = 453	*p* Value
	Preoperative variables			
Age, years	54.00 (44.00–61.00)	50.00 (38.00–60.00)	54.00 (46.00–61.00)	0.0486
Male gender	382 (72.4)	54 (72)	328 (72.4)	0.9419
BMI	26.40 (23.65–29.45)	26.20 (21.50–28.80)	26.40 (23.90–29.50)	0.4681
MELD	22.00 (19.00–23.00)	27.00 (22.00–36.00)	22.00 (18.00–22.00)	<0.0001
Blood urea nitrogen	33.50 (24.00–48.00)	64.00 (27.00–129.00)	32.00 (24.00–44.00)	<0.0001
Creatinine	0.92 (0.80–1.30)	1.30 (0.90–3.00)	0.90 (0.70–1.20)	<0.0001
MDRD pre	81.86 (58.78–107.69)	57.74 (21.95–87.80)	84.07 (62.63–108.76)	<0.0001
Total bilirubin, mg/dL	2.80 (1.21–6.79)	6.79 (2.78–16.64)	2.41 (1.10–5.68)	<0.0001
Hepatocarcinoma	194 (36.7)	12 (16)	182 (40.2)	<0.0001
Acute hepatitis	34 (6.4)	19 (25.3)	15 (3.3)	<0.0001
Other diagnoses	294 (56.32)	44 (14.97)	250 (85.03)	0.6582
	**Intraoperative variables**			
Surgery duration, hours	6.00 (5.00–7.00)	7.00 (5.00–8.00)	6.00 (5.00–7.00)	0.0306
Intraoperative bypass	89 (16.9)	17 (22.7)	72 (15.9)	0.1467
No intraoperative transfusion	131 (24.8)	8 (10.7)	123 (27.15)	0.0022
Platelet transfusion, yes/no	107 (20.27)	31 (41.3)	76 (16.8)	<0.0001
Platelets, mL	300.00 (260.00−500.00)	400.00 (260.00−500.00)	276.50 (260.00−500.00)	0.1053
Plasma transfusion, yes/no	321 (60.80)	63 (84)	258 (57)	<0.0001
Plasma, units	5.00 (3.00–8.00)	8.00 (5.00–12.00)	4.00 (2.00–6.00)	<0.0001
PRBC transfusion, yes/no	355 (67.23%)	65 (86.7)	290 (64.1)	0.0001
PRBC, units	4.00 (2.00–7.00)	7.00 (4.00–12.00)	4.00 (2.00–6.00)	<0.0001
Intraoperative use of noradrenaline, *n* (%)	258 (48.86%)	43 (57.3)	215 (47.5)	0.1131
Noradrenaline maximum dosage, mcg/kg/min	0.20 (0.10–0.30)	0.30 (0.15–0.50)	0.15 (0.10–0.25)	<0.0001
	**Outcomes**			
ICU length of stay, days	2 (1–5)	10 (5–21)	2 (1–4)	<0.0001
Hospital length of stay, days	17.00 (12–32)	37 (20–74)	16 (11–27)	<0.0001
Deceased at 1 year	73 (13.8)	40 (53.3)	33 (7.3)	<0.0001

**Table 2:** Logistic regression analysis of all preoperative and intraoperative factors. MELD: Model for End-stage Liver Disease; MDRD: Modification of Diet in Renal Disease; PRBC: packed red blood cells.

	Odds Ratio (OR)	Confidence Interval (CI)	*p* Value
Male vs. Female	1.117	0.665–1.876	0.6758
Hepatocarcinoma	0.960	0.597–1.543	0.8663
Acute	1.569	0.686–3.589	0.2861
Other	0.917	0.579–1.454	0.7126
Age	1.018	1.002–1.034	0.0267
Body mass index	0.984	0.916–1.056	0.6468
MELD	1.044	1.010–1.078	0.0095
Blood urea nitrogen	1.008	1.003–1.013	0.0023
Creatinine	1.135	0.979–1.315	0.0941
MDRD pre	0.996	0.993–1.000	0.0475
Total Bilirubin	1.024	1.001–1.046	0.0375
Surgery duration	1.055	0.973–1.144	0.1941
Platelets unit (just transfused: *n* = 107)	1.001	0.998–1.003	0.6011
Plasma unit (just transfused: *n* = 321)	1.070	1.010–1.135	0.0223
PRBC units (just transfused: *n* = 355)	1.046	0.999–1.095	0.0542
Hypotension	1.022	1.005–1.040	0.0110
Noradrenaline max dose, 0.1 increase	0.906	0.663–1.237	0.5330

**Table 3:** Odds ratios, confidence intervals and *p*-values for predictive variables are shown. BMI: Body mass index; OLT: orthotopic liver transplantation; MDRD: Modification of Diet in Renal Disease; PRBC: packed red blood cells.

	Odds Ratio (OR)	Confidence Interval (CI)	*p* Value
Male vs. Female	0.980	0.568–1.689	0.9417
Hepatocellular carcinoma	0.284	0.149–0.541	0.0001
Acute	9.907	4.765–20.596	<0.001
Other	1.118	0.681–1.837	0.6583
End stage liver disease	2.780	0.834–9.266	0.0961
Alcohol	0.570	0.237–1.370	0.2086
Biliary	0.898	0.367–2.198	0.8134
Other	1.924	1.088–3.404	0.0245
Criptogenetic	0.588	0.175–1.976	0.3901
Bypass	1.551	0.854–2.816	0.1492
Age, year	0.990	0.977–1.003	0.1369
BMI	1.015	0.946–1.088	0.6828
MDRD pre-OLT	1.000	0.998–1.001	0.5062
Surgery duration, hours	1.075	0.988–1.168	0.0914
Platelet, unit	1.002	1.000–1.004	0.0847
Plasma, unit	1.130	1.068–1.195	<0.0001
PRBC, unit	1.142	1.081–1.206	<0.0001
Hypotension	1.018	1.000–1.037	0.0500
Noradrenaline, 0.1 increase	1.076	0.910–1.273	0.3901
Vasopressin, 0.1 unit	1.610	0.802–3.230	0.1802
Creatinine day 1	2.238	1.611–3.109	<0.0001
MDRD day 1	0.999	0.998–1.001	0.3546
Total bilirubin day 1	1.134	1.078–1.193	<0.0001
Tacrolimus day1	0.794	0.559–1.128	0.1975
Platelets day 1	0.988	0.980–0.997	0.0097
Urine output D1	0.998	0.998–0.999	<0.0001

**Table 4:** Factors associated with changes in creatinine over follow-up. Estimate, confidence limits, and *p*-values are shown. MELD: Model for End-stage Liver Disease; PRBC: packed red blood cells; CRRT: continuous renal replacement therapy.

Variable	Estimate Variation	95% Confidence Limits	*p* Value
Female gender	−0.108	−0.243; 0.028	0.120
Age	0.009	0.006; 0.011	<0.001
Body Mass Index	0.012	−0.10; 0.034	0.274
MELD	0.007	−0.002; 0.015	0.114
Preoperative Blood Urea Nitrogen	0.002	−0.001; 0.005	0.097
Preoperative Total Bilirubin	0.001	−0.007; 0.008	0.834
Diagnosis			
-Acute Hepatitis vs. Cirrhosis	−0.020	−0.205; 0.165	0.834
-Hepatocarcinoma vs. Cirrhosis	−0.069	−0.161; 0.022	0.139
Intraoperative Bypass	−0.043	−0.104; 0.181	0.567
Surgery Duration, minutes	0.001	−0.019; 0.021	0.898
Platelet Transfusion, unit	0.148	−0.078; 0.374	0.199
Plasma Transfusion, unit	0.006	−0.008; 0.020	0.422
PRBC Transfusion, unit	0.007	−0.007; 0.022	0.316
Intraoperative Hypotension	−0.008	−0.012; 0.003	<0.001
Noradrenaline maximum dosage, per 0.01 mcg/kg/min increase	0.105	0.053; 0.157	<0.001
CRRT in the first week	0.246	0.056; 0.436	0.011

## Data Availability

The datasets used and analyzed are available from the corresponding author on reasonable request.
